# Supplementary material for: Revealing Physiochemical Factors and Zooplankton Influencing Microcystis Bloom Toxicity in a Large-Shallow Lake Using Bayesian Machine Learning
Source: Toxins (Basel). 2022 Aug 2;14(8):530. doi: 10.3390/toxins14080530 (PMC9413751; doi:10.3390/toxins14080530)
Supplement: Supplementary file 1 [file toxins-14-00530-s001.zip › toxins-1818921-supplementary.pdf]

Supplementary Materials: Revealing Physiochemical Factors and Zooplankton Influencing *Microcystis* Bloom Toxicity in a Large-Shallow Lake Using Bayesian Machine Learning

Xiaoxiao Wang, Lan Wang, Mingsheng Shang, Lirong Song and Kun Shan

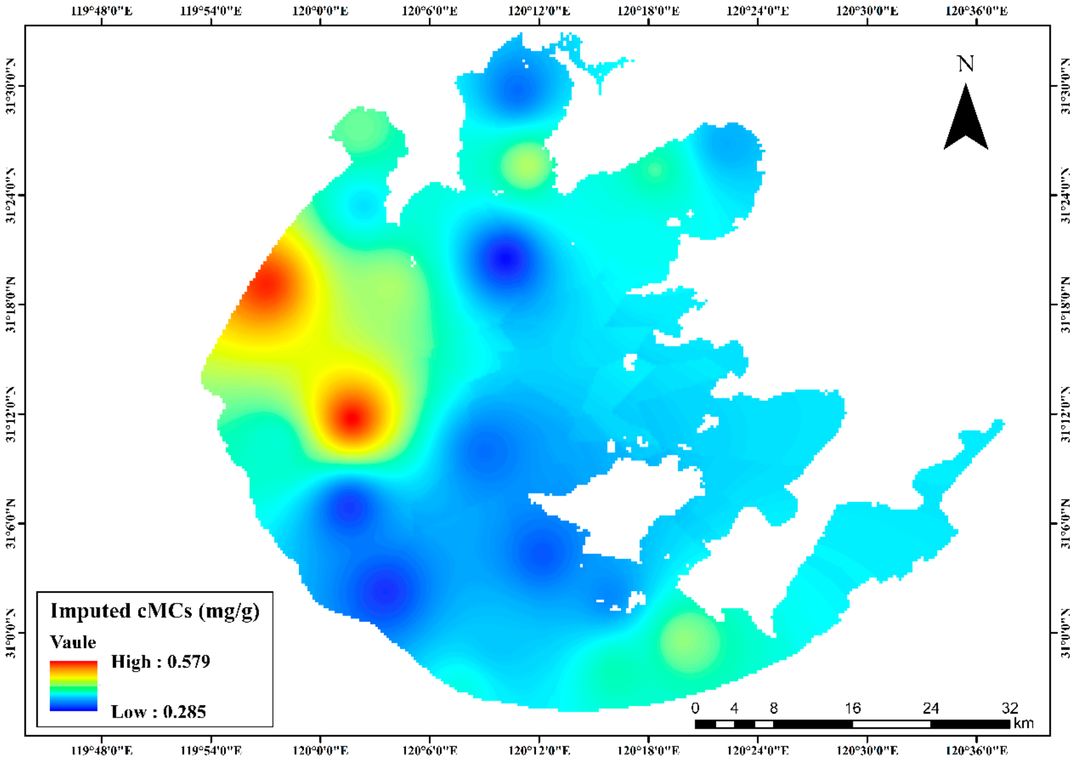

Figure S1. The distributions of the annual mean of intracellular microcystins (cell-bound MCs) across 22 sampling sites in Lake Taihu based on NLF imputation.

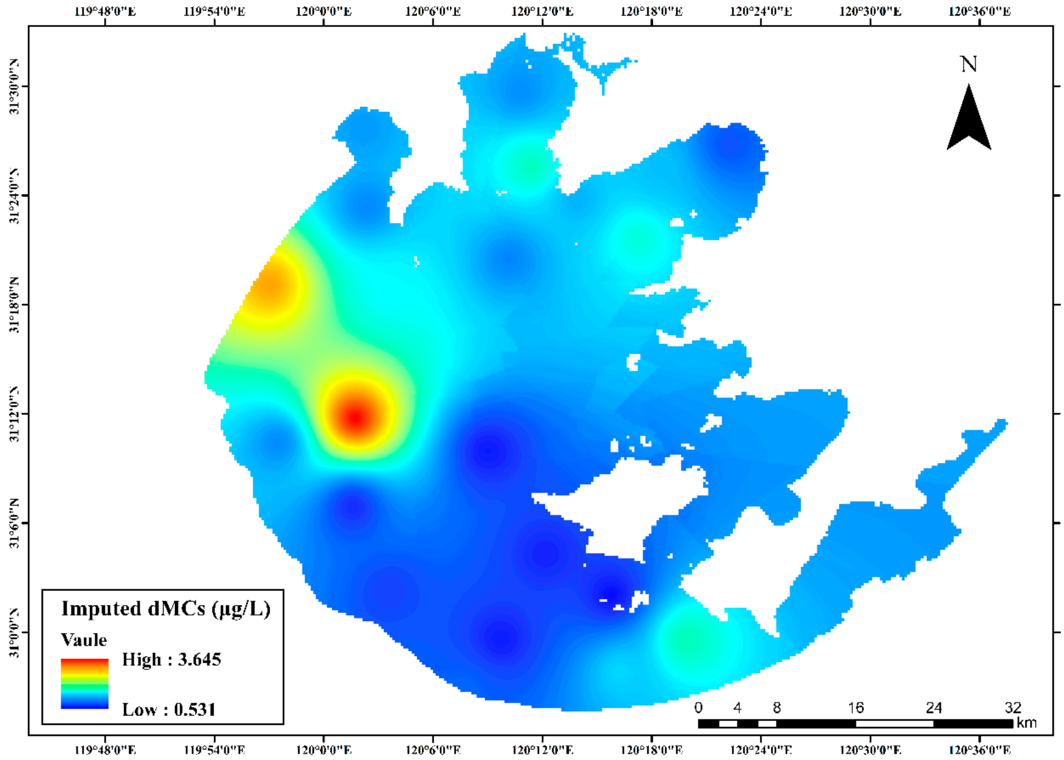

Figure S2. The distributions of the annual mean of extracellular microcystins (dissolved MCs) across 22 sampling sites in Lake Taihu based on NLF imputation.

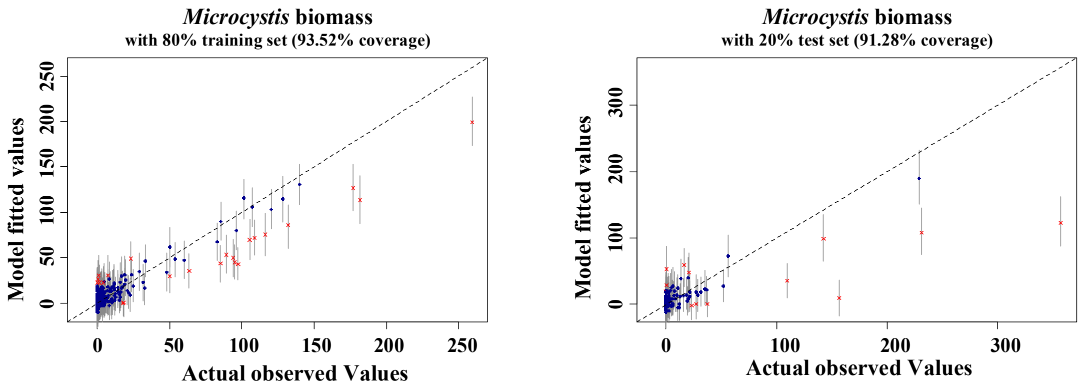

Figure S3. Accuracy validation of the BART model for *Microcystis* biomass with 95% confidence interval for training set (Left) and test set (Right).

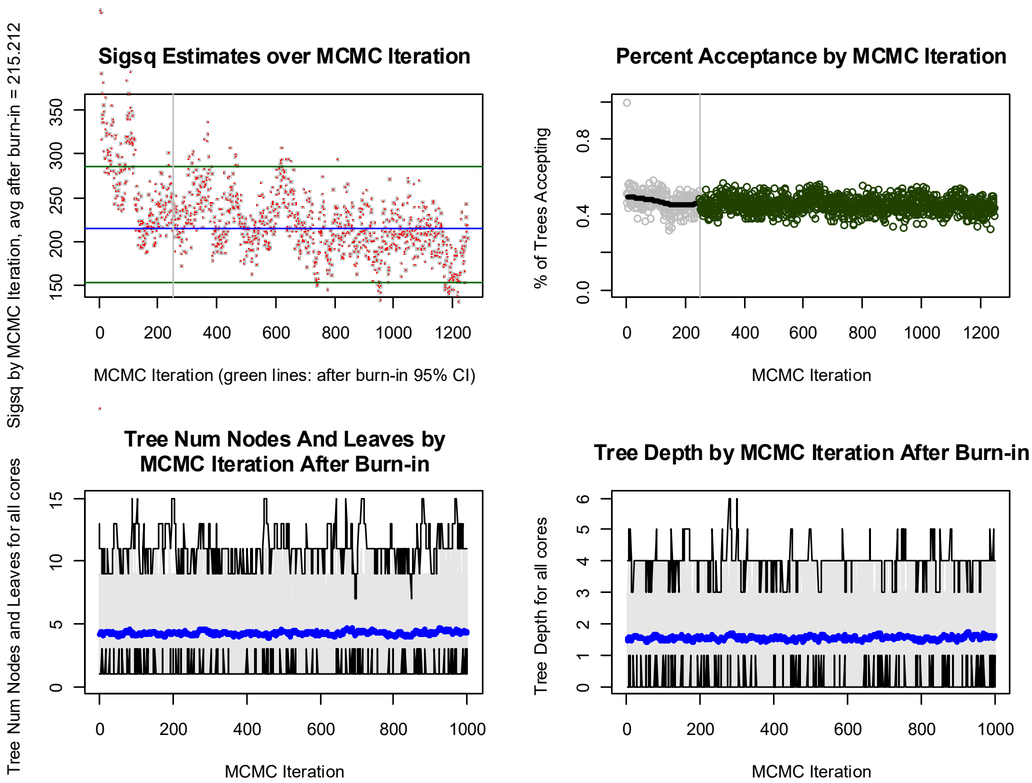

Figure S4. Convergence statistics of the NLF-BART model for *Microcystis* biomass prediction. (a): Sigsq denotes the variance. (b): Acceptance rate of Metropolis-Hastings sampling over the trees. (c): Average number of nodes and leaves over m trees after burn-in. (d): Average tree depth over m trees.

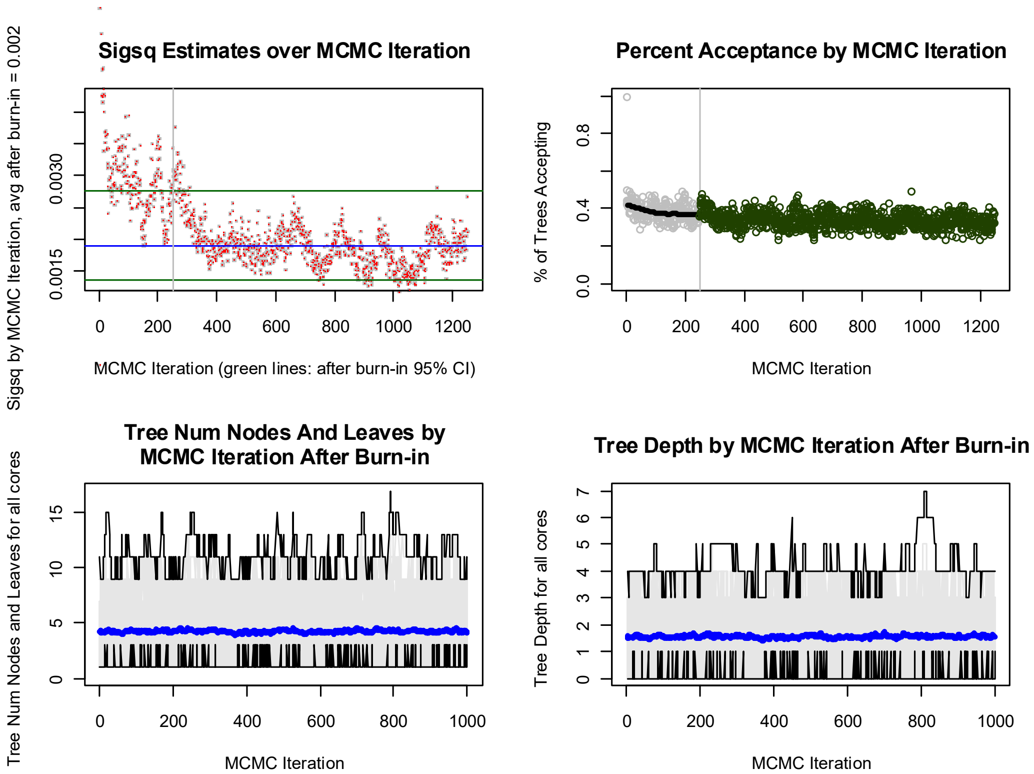

Figure S5. Convergence statistics of the NLF-BART model for intracellular MCs prediction.

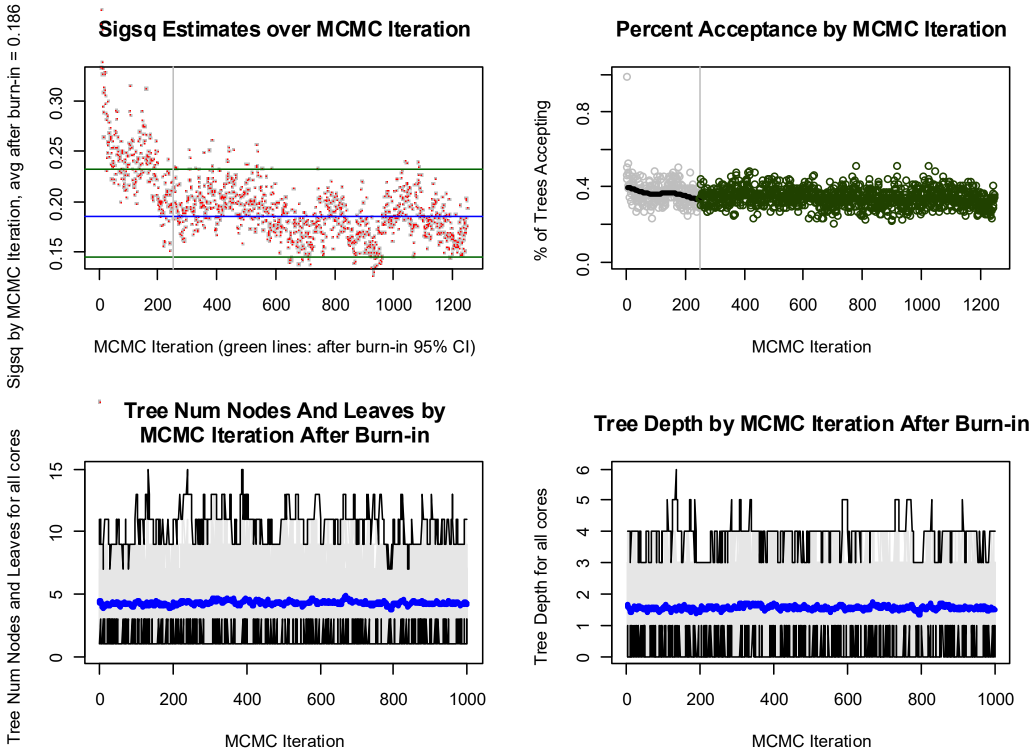

Figure S6. Convergence statistics of the NLF-BART model for extracellular MCs prediction.
